# Trends regarding the profile of cardiac surgery patients during the first wave of COVID-19 pandemic in Greece

**DOI:** 10.21470/1678-9741-2021-0015

**Published:** 2022

**Authors:** Antonios Roussakis, Konstantinos Boumpoulis, Ioannis Nenekidis, Aikaterini Gavalaki, Konstantinos Petsios, Stavros Dimopoulos, Ioannis Bisiadis, Panagiota Rellia, Konstantinos Perreas

**Affiliations:** 1 1^st^ Cardiac Surgery Department, Onassis Cardiac Surgery Center, Athens, Greece. antonisroussakis@yahoo.gr; 2 Clinical Research Office, Onassis Cardiac Surgery Center, Athens, Greece.; 3 Intensive Care Unit, Onassis Cardiac Surgery Center, Athens, Greece.; 4 Anesthesiology Department, Onassis Cardiac Surgery Center, Athens, Greece.

Humanity faces one of the greatest healthcare challenges ever, due to the COVID-19 pandemic. According to WHO’s weekly operational report, published on 16^th^ February 2021, we have 110,384,747 confirmed cases and 2,446,008 deaths globally (https://www.who.int/emergencies/diseases/novel-coronavirus-2019/situation-reports). SARS-CoV-2 has been highly infectious, and it did not take long to cross the borders of China and soon become a global health threat with implications of unpredictable magnitude^[1]^. Noticeably, the rate of new infections is still high, with more than 2.7 million new cases per week, and there is a great concern regarding public health events associated with SARS-CoV-2 variants.

Concerning cardiac surgery units, there was a clear impact on the daily routine in different ways^[2-5]^. Many changes took place regarding admission procedures, aggressive infection mitigation strategies in the operating room and during surgical recovery, intensive care guidelines and restrictions. Also, a necessity rose to postpone elective cardiac surgeries, to manage healthcare workers’ resources differently, to deal with the increased risk of infection for both patients and health professionals, along with the exaggerated skepticism among patients, affecting their willingness to undergo a cardiac surgery. In an international survey during the first wave of the pandemic, with the participation of more than 600 cardiac surgeons from America, Europe, Asia, and Australia, a median reduction in the volume of cardiac surgery cases was 50% to 75%, as most centers postponed elective cases and more than one-third of the centers reported more than 50% reductions in intensive care capacity^[6]^. This led to modifications in policies and guidelines regarding cardiac surgery priority to confront the pandemic requirements and scarce resources. However, these recommendations should be considered on case-by-case basis with a clear need for regular updates^[7-9]^.

The Onassis Cardiac Surgery Center (OCSC) is a tertiary hospital focused on the treatment of cardiac surgery and cardiology patients. The core period of the first wave of the pandemic in Greece was the trimester from March to May 2020, with the peak of the crisis occurring in April^[10]^. During this period, some interesting changes in the characteristics of the patients treated by the 1^st^ Cardiac Surgery Department were noted. Comparative demographic data between 2018 and 2020 for the patients operated in April of each year are presented in Table 1.

**Table 1:** Demographic characteristics of the study population.

Demographic characteristics	April 2018 (N=43)	April 2019 (N=52)	April 2020 (N=50)	Total sample (N=145)
Age*	69.05±9.52 (69)	67.92±12.63 (72)	71.04±7.14 (73)	69.33±10.1 (72)
Sex				
Male	11 (25.6%)	14 (26.9%)	17 (34%)	42 (29%)
Female	32 (74.4%)	38 (73.1%)	33 (66%)	103 (71%)
EuroSCORE II	2.45±2.48 (1.75)	2.37±2.69 (1.58)	3.92±2.26 (1.93)	2.93±5.30 (1.75)
CCS				
Unspecified	0 (0%)	0 (0%)	5 (10%)	5 (3.4%)
Type I	12 (27.9%)	10 (19.2%)	7(14%)	29 (20%)
Type II	10 (23.3%)	7 (13.5%)	13 (26%)	30 (20.7%)
Type III	1 (2.3%)	1 (1.9%)	1 (2%)	3 (2.1%)
Type IV (unstable)	0 (0%)	1 (1.9%)	1 (2.0%)	2 (1.4%)
No angina	20 (46.5%)	33 (63.5%)	23 (46%)	76 (52.4%)
NYHA				
Type I	10 (23.3%)	17 (32.7%)	8 (16%)	35 (24.1%)
Type II	29 (67.4%)	29 (55.8%)	34 (64%)	90 (62.1%)
Type III	3 (7.0%)	6 (11.5%)	8 (16%)	17 (11.7%)
Type IV	1 (2.3%)	0 (0%)	2 (4%)	3 (2.1%)
History of MI	13 (30.2%)	11 (21.2%)	17 (34%)	41 (28.3%)
Duration of mechanical ventilation (hours)	101.2±235.5 (56)	82.44±156 (40)	70.76 ± 87.2 (47)	83.99±166.1 (40)
Duration of ICU care (hours)	48.3±231.1 (8)	34.7±132.3 (8)	23.1±38.1 (14)	34.74 ±149.6 (8)
Duration of postoperative hospitalization (days)	9.58±9.74 (7)	9.01±8.07 (7)	8.34±3.68 (7)	8.94±7.46 (7)

* Mean±SD (median). CCS=Canadian Cardiovascular Society; ICU=intensive care unit; MI=myocardial infarction; NYHA=New York Heart Association

**Fig. 1:**
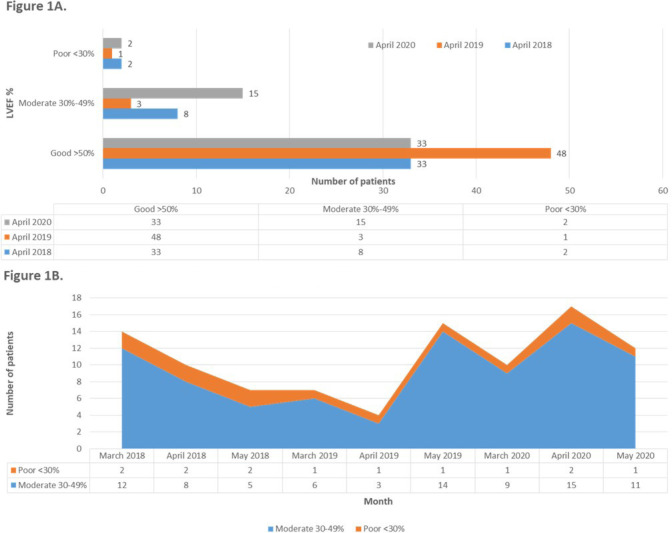
(A) Comparison of LVEF categories. (B) Patients with LVEF <50% (March-April-May 2018-2020).

The left ventricular ejection fraction (LVEF) of patients operated in April 2020 was found statistically lower in comparison to previous years (*P*=0.024, df=4, Pearson’s chi-square=11.209) (Figure 1A). When comparing all 3 months of each year, only a clear trend was detected towards LVEF<50% for the COVID-19 period (*P*=0.075, df=4, Pearson’s chi-square=6.342) (Figure 1B). The mean EuroSCORE II of patients operated in April 2021 was considered elevated, even though this increase was marginally not statistically significant (*P*=0.067, df=143, t=1.664).

In an attempt to explain this finding and taking under consideration the facts of Greek reality during this health crisis, we were driven to a multifactorial hypothesis. The strict and prolonged quarantine limited drastically the physical activity of cardiac patients and their exposure to environmental triggers, leading to a decrease in the onset of new cardiac related symptoms or a limitation in the repetition of known ones, creating a “pseudo-asymptomatic” group of patients. Furthermore, it was noticed that even symptomatic patients delayed significantly to seek medical help due to worry or “fear” of exposing themselves to the risk of SARS-CoV-2 infection^[11,12]^. Moreover, in some cases, wasted valuable time was detected through the procedure of referrals from referring cardiology clinics to hospitals with cardiac surgery service. In addition, a number of centers postponed elective cardiac operations and even shifted their services as the special conditions demanded during the crisis. However, in our case, the strict safety policy undertaken from our hospital, with real-time PCR for COVID-19 contacted prior to the admission of all patients, along with other preventive measures, may have positively affected patients' sense of security and influenced their decision not to postpone their surgery. In accordance, Shehata et al.^[13]^ stated that, even though cardiac patients are at higher risk of developing COVID-19 in the perioperative period, the implementation of effective measures have been proved to prevent nosocomial transmission of COVID-19. Moreover, admission and provision of care to a non-COVID-19 unit, with a strict policy for use of personal protective equipment (PPE) and hand hygiene, is safer than being in the community, especially in periods of increased incidence. The magnitude of the impact of the above observations on the clinical status or prognosis of the patients remains unknown and, to investigate it thoroughly, multicenter studies are needed to combine and compare their experience in the subject.

Our study has several limitations. Our results can be comparable to settings where infection rates remained similarly low to Greece and the time of surgical delay was not extremely prolonged. Our hospital policy enabled our department to be fully active and to perform cardiac surgery operations during the entire period of the pandemic. This was in accordance with the recommendations of the Greek Ministry of Health. However, the management followed by other hospitals may differ from OCSC’s.

In conclusion, the COVID-19 pandemic has resulted in considerable delays and affected the patients’ clinical profile due to the implementation of restrictive measures in cardiac surgery units. Even though these medical collateral damages are still in the shadows, when the pandemic is managed, they will fully emerge. Therefore, this type of study is essential to implement a strategic plan for the management of cardiac surgery patients in the COVID-19 era, by acknowledging the possibility of prolonged recovery and the trends regarding elective or emergency operations based on healthcare professionals’ decisions and patient willingness to attend the hospital for surgery. Communication policies to prevent medical avoidance behavior are recommended, along with a clear policy regarding selection criteria for elective and emergency cardiac operations.
